# Proteomic analysis of enterotoxigenic *Escherichia coli* (ETEC) in neutral and alkaline conditions

**DOI:** 10.1186/s12866-016-0914-1

**Published:** 2017-01-07

**Authors:** Lucia Gonzales-Siles, Roger Karlsson, Diarmuid Kenny, Anders Karlsson, Åsa Sjöling

**Affiliations:** 1 Department of Infectious Disease, Institute of Biomedicine, Sahlgrenska Academy, University of Gothenburg, SE-41346 Gothenburg, Sweden; 2 Nanoxis Consulting AB, SE-40016 Gothenburg, Sweden; 3 Proteomics Core Facility at the University of Gothenburg, SE-43050 Gothenburg, Sweden; 4 Department of Microbiology Tumor and Cell Biology, Karolinska Institutet, Stockholm, SE-17177 Sweden

**Keywords:** ETEC, pH regulation, Proteomics, Alkaline, ATPase, OmpA, BAM

## Abstract

**Background:**

Enterotoxigenic *Escherichia coli* (ETEC) is a major cause of diarrhea in children and travelers to endemic areas. Secretion of the heat labile AB_5_ toxin (LT) is induced by alkaline conditions. In this study, we determined the surface proteome of ETEC exposed to alkaline conditions (pH 9) as compared to neutral conditions (pH 7) using a LPI Hexalane FlowCell combined with quantitative proteomics. Relative quantitation with isobaric labeling (TMT) was used to compare peptide abundance and their corresponding proteins in multiple samples at MS/MS level. For protein identification and quantification samples were analyzed using either a 1D-LCMS or a 2D-LCMS approach.

**Results:**

Strong up-regulation of the ATP synthase operon encoding F1Fo ATP synthase and down-regulation of proton pumping proteins NuoF, NuoG, Ndh and WrbA were detected among proteins involved in regulating the proton and electron transport under alkaline conditions. Reduced expression of proteins involved in osmotic stress was found at alkaline conditions while the Sec-dependent transport over the inner membrane and outer membrane protein proteins such as OmpA and the β-Barrel Assembly Machinery (BAM) complex were up-regulated.

**Conclusions:**

ETEC exposed to alkaline environments express a specific proteome profile characterized by up-regulation of membrane proteins and secretion of LT toxin. Alkaline microenvironments have been reported close to the intestinal epithelium and the alkaline proteome may hence represent a better view of ETEC during infection.

## Background

Enterotoxigenic *Escherichia coli* (ETEC) remains to be one of the major causes of childhood diarrhea and is a global health problem [1]. ETEC cause disease by adhering to the epithelium of the small intestine by means of different colonization factors [2]. The two major ETEC toxins, heat labile toxin (LT) and heat stable toxin (ST), binds to enteric receptors on the epithelium and ultimately cause de-regulation of the chloride channel CFTR, which leads to increased secretion of chloride ions, bicarbonate and electrolytes [3]. LT is an AB_5_ toxin encoded by the *eltA* and *eltB* genes in one operon. The LTA and LTB peptides are secreted through sec dependent mechanisms to the periplasm and assembled by DsbA [4]. Secretion through the outer membrane occurs via the Type II secretion system (T2SS) [5]. Secretion of LT has been reported to vary between ETEC isolates, ranging from being completely retained in the periplasm [6], to secretion of up to 50% of the produced LT holotoxin in LB media [7–9]. The ST toxin is also transported in a Sec-dependent manner through the inner membrane but is released through TolC [10]. The small ST peptide is cleaved and folded in the process and the mature peptide is secreted to the outer environment.

ETEC encounter different environments in the human gastrointestinal tract before reaching optimal conditions for infection in the small intestine and environmental cues, such as bile, oxygen and pH affect secretion of toxins and virulence of ETEC [7, 11, 12]. Passage through the stomach exposes infecting pathogens to acidic conditions, while entry into the duodenum is characterized by a rise of pH due to release of bile and bicarbonate [13, 14]. Further down in the anaerobic gut the pH is expected to drop to acidic levels again but close to the small intestinal epithelium alkaline conditions can occur due to release of bicarbonate. Alkaline surface microclimates in the small intestine have been described previously [15]. ETEC toxins ST and LT both enhance secretion of bicarbonate through activation of the CFTR ion channel, which might create an extremely alkaline microenvironment close to the infecting bacteria. Interestingly, similar to the highly homologous cholera toxin (CT) the assembly of LT seems to be dependent on an alkaline environment [7, 16, 17]. We have previously shown that secretion of LT toxin is favored under alkaline conditions and inhibited under acidic conditions [7]. Hence our results support the hypothesis that ETEC toxin secretion is induced at alkaline conditions at the site of infection. In this study we analyzed the proteome of ETEC exposed to alkaline conditions (pH 9) as compared to neutral conditions (pH 7) in order to further determine the effect of highly alkaline conditions on ETEC.

## Methods

### Overview of methodology

**Fig. 1:**
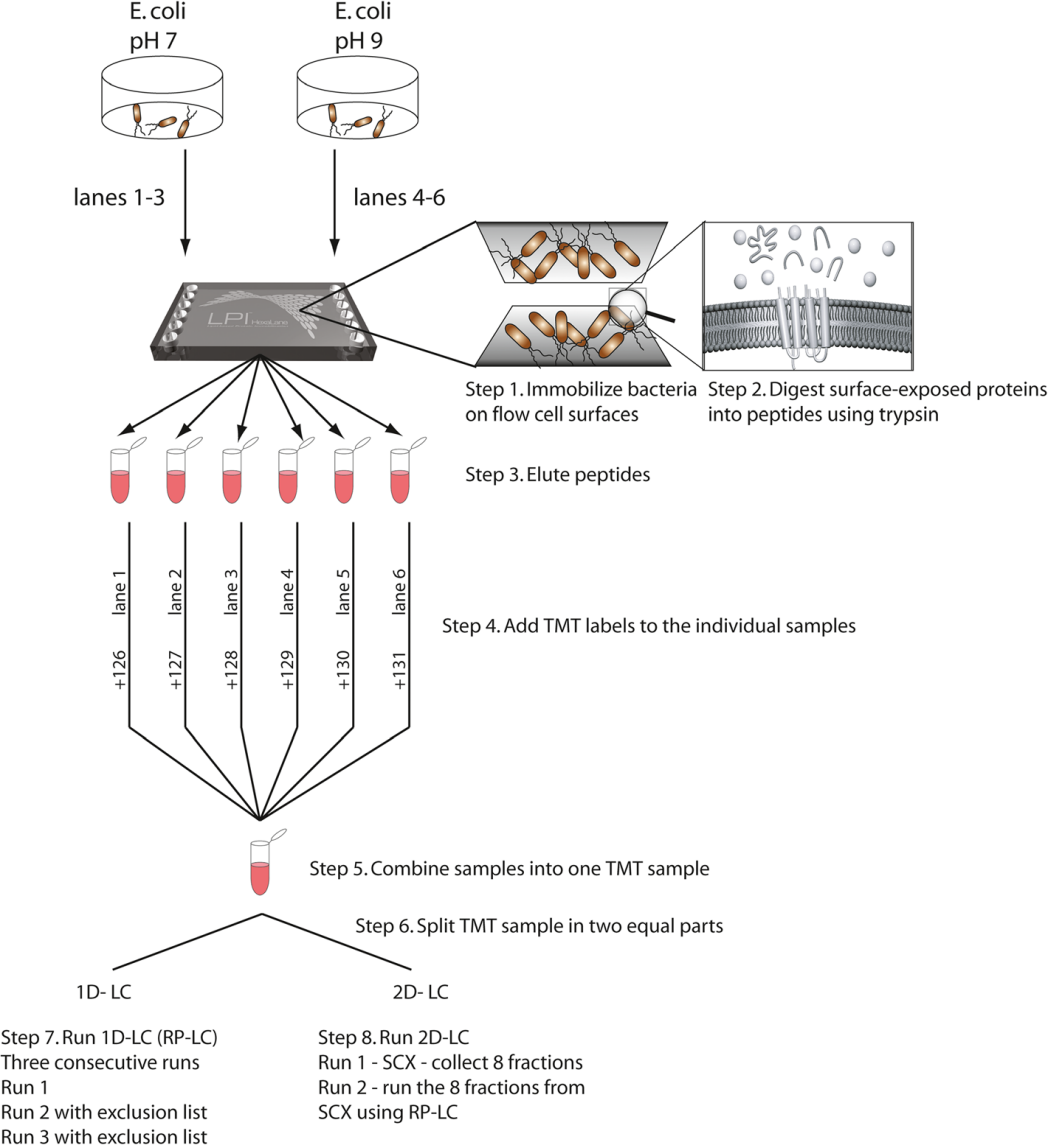
Overall workflow of the methodology applied in the study. Three independent TMT sets were analyzed from three biological replicates, grown and analyzed at different time points

Clinical isolate ETEC E2863 was cultured in either pH 7 or pH 9 LBK media at three separate occasions to produce three biological replicates. For each biological replicate, we include three technical replicates. The bacteria culture for each pH condition was immobilized and digested in three separate LPI Hexalane channels generating three separate peptide samples (Fig. 1). Peptide samples generated for both pH conditions were labelled with the TMT (6-plex) kit and combined into one set. The set was then split into two aliquots for analysis with either 1D-LC or 2D-LC fractionation followed by MS analysis (Fig. 1). Following MS analysis and database matching relative quantification was performed. Proteins displaying more than 20% variation between the three samples from the individual LPI channels at each condition were removed. This was done by calculation the ratio of the separate TMT-labels in a group, and the average of the combined channels e.g. 126/(average 126 + 127 + 128). Proteins with rations between 0.8 and 1.2 were included in the protein list. For comparison of the two conditions, fold changes were calculated and a statistical analysis Welch’s *t*-test was used for multiple comparisons. Only proteins passing the statistical filter (*p* < 0.05) were accepted. Additionally, all three biological replicates, were statistically evaluated as described above resulting in three separate lists of quantified proteins considering a fold change of at least 1.5 as a threshold for considering relevant up or down regulation. Finally, the proteins accepted for the biological interpretation were quantified in at least two of the three TMT-sets and biological replicates.

### Culture conditions

The ETEC clinical isolate E2863 was used in the study. E2863 was grown in LBK media (10 g Tryptone, 5 g yeast extract, 6.4 g KCl) buffered to pH 7 using piperazine-N, N9-bis-(2-ethanesulfonic acid) (PIPES) or pH 9 using 3-[(1,1-dimethyl-2-hydroxyethyl)amino]-2-hydroxypropanesulfonic acid (AMPSO) at 100 mM. Media were adjusted for pH with KOH, to avoid high concentrations of sodium ions, which inhibit growth at high pH. These buffers help cultures to maintain a constant pH throughout growth. All cultures were grown for 3 h since the highest secretion levels of LT toxin has been reported to occur at this time [7], pH 7 cultures reached an OD_600_ of 1.2 whereas pH 9 cultures reached an OD_600_ of 0.4.

### Trypsin digestion of bacteria in LPI HexaLane FlowCell and TMT (tandem mass tags) labeling

The bacterial biomass was washed with PBS three consecutive times by centrifugation of the samples for 5 min at 10.000 rpm, followed by discarding the supernatant and then resuspending the pellet in 1 ml PBS. The washed bacterial suspension was injected into the LPI Hexalane FlowCell (Nanoxis Consulting AB, www.nanoxisconsulting.com) by adding 100 μL to fill the FlowCell channel (with a volume of ∼ 30 μL) using a pipette. The excess of bacterial suspension was removed from the inlet and outlet ports. The immobilized bacteria were incubated for 1 h, at room temperature, to allow bacterial cell attachment, and the FlowCell channels were washed subsequently with 1.0 mL of TEAB (Triethylammonium bicarbonate) using a syringe pump, with a flow rate of 100 μL/min. Enzymatic digestion of the bacterial proteins was performed by injecting 100 μL of trypsin (20 μg/mL in 200 mM TEAB, pH ~8) into the FlowCell channels and incubating for 30 min at room temperature. The generated peptides were eluted by injecting 200 μl TEAB (200 mM, pH ~8) into the FlowCell channels at a flow rate of 100 μL/min. The eluted peptides were collected at the outlet ports, using a pipette, and transferred into Axygen tubes (2 ml). The peptide solutions were incubated at room temperature overnight, to allow for complete digestion, and subsequently frozen at −20 °C. As described above, each of the three biological replicates at both conditions were analyzed using triplicate samples of pH 7 and pH 9 (technical replicates) in order to allow for technical variation and to give statistical support for the *t*-test analysis.

The digested samples were concentrated to 30 μl and 70 μl of 0.5 M TEAB (Triethylammonium Bicarbonate) was added to each tube prior to labeling with the TMT® according to the manufacturer’s instructions (Thermo Scientific). In a set, each sample was labeled with a unique tag from a TMT 6plex isobaric mass tag labeling kit. After TMT labeling, the samples in a set were pooled resulting in three independent sets in total to cover all samples.

### LC-MS/MS Analysis on LTQ-Orbitrap Velos and Q-Exactive

Each set was divided in two equal volumes into two separate samples (sample 1 and sample 2) that were either subjected to LCMS-analysis directly (1D-LC) or further purified and fractionated by Strong Cation Exchange Chromatography (SCX) followed by LCMS-analysis (2D-LC). Sample 1, analyzed according to the 1D-LC approach, was desalted using PepClean C18 spin columns (Thermo Fisher Scientific) according to the manufacturer’s guidelines prior to LCMS-analysis. The second sample (sample 2) was fractionated using SCX spin columns (Thermo Fisher Scientific) into 8 fractions according to the manufacturer’s guidelines followed by a desalting step of each fraction.

Samples were reconstituted with 15 μl of 0.1% formic acid (Sigma Aldrich) in 3% acetonitrile (Sigma Aldrich) and analyzed on either an LTQ-Orbitrap Velos or Q-exactive (Thermo Fisher Scientific, Inc., Waltham, MA, USA) mass spectrometer interfaced to an Easy-nLC II (Thermo Fisher Scientific). Peptides (2 μL injection volume) were separated using an in-house constructed analytical column (200 × 0.075 mm I.D.) packed with 3 μm Reprosil-Pur C18-AQ particles (Dr. Maisch, Germany). Solvent A was 0.2% formic acid in water and solvent B was 0.2% formic acid in acetonitrile. The following gradient was run at 200 nL/min; 5–30% B over 75 min, 30–80% B over 5 min, with a final hold at 80% B for 10 min. Ions were injected into the mass spectrometer under a spray voltage of 1.6 kV in positive ion mode. The MS scans was performed at 30 000 and 70 000 resolution (at *m/z* 200) with a mass range of *m/z* 400–1800 for the Velos and Q-exactive, respectively. MS/MS analysis was performed in a data-dependent mode, with the top ten most abundant doubly or multiply charged precursor ions in each MS scan selected for fragmentation (MS/MS) by stepped high energy collision dissociation (stepped HCD) of NCE-value of 25, 35 and 45. For MS/MS scans the resolution was 7 500 and 35,000 (at *m/z* 200) for the Velos and Q-exactive with a mass range of *m/z* 100–2000. The isolation window was set to 1.2 Da, intensity threshold of 1.1e4 and a dynamic exclusion of 30 s, enabling most of the co-eluting precursors to be selected for MS/MS. Samples analyzed according to the 1D-LC approach were re-analyzed twice with exclusion lists generated after database searching of previous LCMS runs (see below).

### Database search for protein TMT quantification

For relative quantification and identification the MS raw data files for each TMT set were merged in the search using Proteome Discoverer version 1.4 (Thermo Fisher Scientific). For the 1D-LC and 2D-LC approaches, the triplicate injections and the SCX fraction were combined, respectively. A database search for each set was performed with the Mascot search engine (Matrix Science LTD) using species-specific databases downloaded from Uniprot. The data was searched with MS peptide tolerance of 10 ppm for Orbitrap Velos and 5 ppm for Q-Exactive runs and MS/MS tolerance for identification of 100 millimass units (mmu). Tryptic peptides were accepted with 1 missed cleavage and variable modifications of methionine oxidation, cysteine methylthiolation and fixed modifications of N-terminal TMT6plex and lysine TMT6plex were selected. The detected peptide threshold in the software was set to 1% FDR (false discovery rate) for the experiments performed on the QExactive, and 5% FDR for the experiments performed on the Velos, by searching against a reversed database. Identified proteins were grouped by sharing the same sequences to minimize redundancy. For the 1D-LC approach exclusion lists of m/z values of the identified peptides with a two minutes retention time window was generated from the search results.

For TMT quantification, the ratios of the TMT reporter ion intensities in MS/MS spectra (m/z 126–131) from raw data sets were used to calculate fold changes between samples. Ratios were derived by Proteome Discoverer using the following criteria: fragment ion tolerance as 80 ppm for the most confident centroid peak and missing values are replaced with minimum intensity. TMT reagent purity corrections factors are used and missing values are replaced with minimum intensity. Only peptides unique for a given protein were considered for relative quantitation, excluding those common to other isoforms or proteins of the same family. The quantification was normalized using the protein median. The results were then exported into MS Excel (Microsoft, Redmond, WA) for manual data interpretation and statistical analysis. Only peptides unique for a given protein were considered for relative quantitation, excluding those common to other isoforms or proteins of the same family.

### Statistical analysis

First, proteins displaying more than 20% variation between the individual LPI channels for the three pH 7 and the three pH 9 channels respectively were removed. This was done by calculation the ratio of the separate TMT-labels in a group, and the average of the combined channels e.g. 126/(average 126 + 127 + 128). Proteins with ratios between 0.8 and 1.2 were included in the protein list. Second, a Welch’s *t*-test was performed (3 technical replicates pH 7 vs 3 technical replicates pH 9) and only proteins passing filter *p* < 0,05 was accepted. Third, a fold change of at least 1.5 was set as a threshold to list proteins that had a relevant up or down regulation. Fourth, the proteins accepted for the biological interpretation had to be quantified in at least two of the three TMT-sets (biological replicates).

## Results

### Surface proteome analysis and protein annotation

To study the effect of alkaline pH on ETEC strain E2863 we used a MS-based quantitative proteomic strategy. Three biological replicates of the experiments were performed in pH 7 and pH 9, respectively. Tandem mass tag (TMT) labeling was used for multiplexed relative quantification of proteins in multiple samples [18]. Since we were interested in the bacterial surface proteome exposed to the environment during alkaline conditions we used the LPI methodology for surface shaving of bacteria to enrich for surface proteome [19].

The peptides generated by the LPI methodology were analyzed with two different separation strategies prior MS analysis to increase the number of detected proteins. Therefore, after eluting peptides from the LPI flow cell the combined sample was split into two equal parts (sample 1 and 2) and analyzed by either an one-dimensional (1D-LC) approach or a two-dimensional (2D-LC) approach including an offline strong cation exchange fractionation step prior to MS-analysis. The overall workflow is depicted in Fig. 1.

Since ETEC strain E2863 is not whole genome sequenced, a proteomic strain typing according to Karlsson et al. was performed*,* to identify the most similar strain to E2863 for peptide matching [19]. Strain identity typing identified *E. coli* K011FL as the top ranking identity strain and it was used for peptide matching. In order to pick up ETEC specific genes, the ETEC reference strain H10407 was used. For each experiment the resulting protein matches using both K011FL and H10407 were annotated and finally all results obtained in the three independent replicates were combined (Table 1).

**Table 1:** Protein matches to genomes used for matching peptides before and after *t*-test analysis (*p* < 0.05)

	K011FL				H10407			
	Total number of proteins		Significant (*p* < 0.05)		Total number of proteins		Significant (*p* < 0.05)	
	1D-LC	2D-LC	1D-LC	2D-LC	1D-LC	2D-LC	1D-LC	2D-LC
Replicate 1	435	470	211	238	440	471	210	240
Replicate 2	579	455	272	200	574	447	264	188
Replicate 3	503	402	292	228	500	413	289	230

**Fig. 2:**
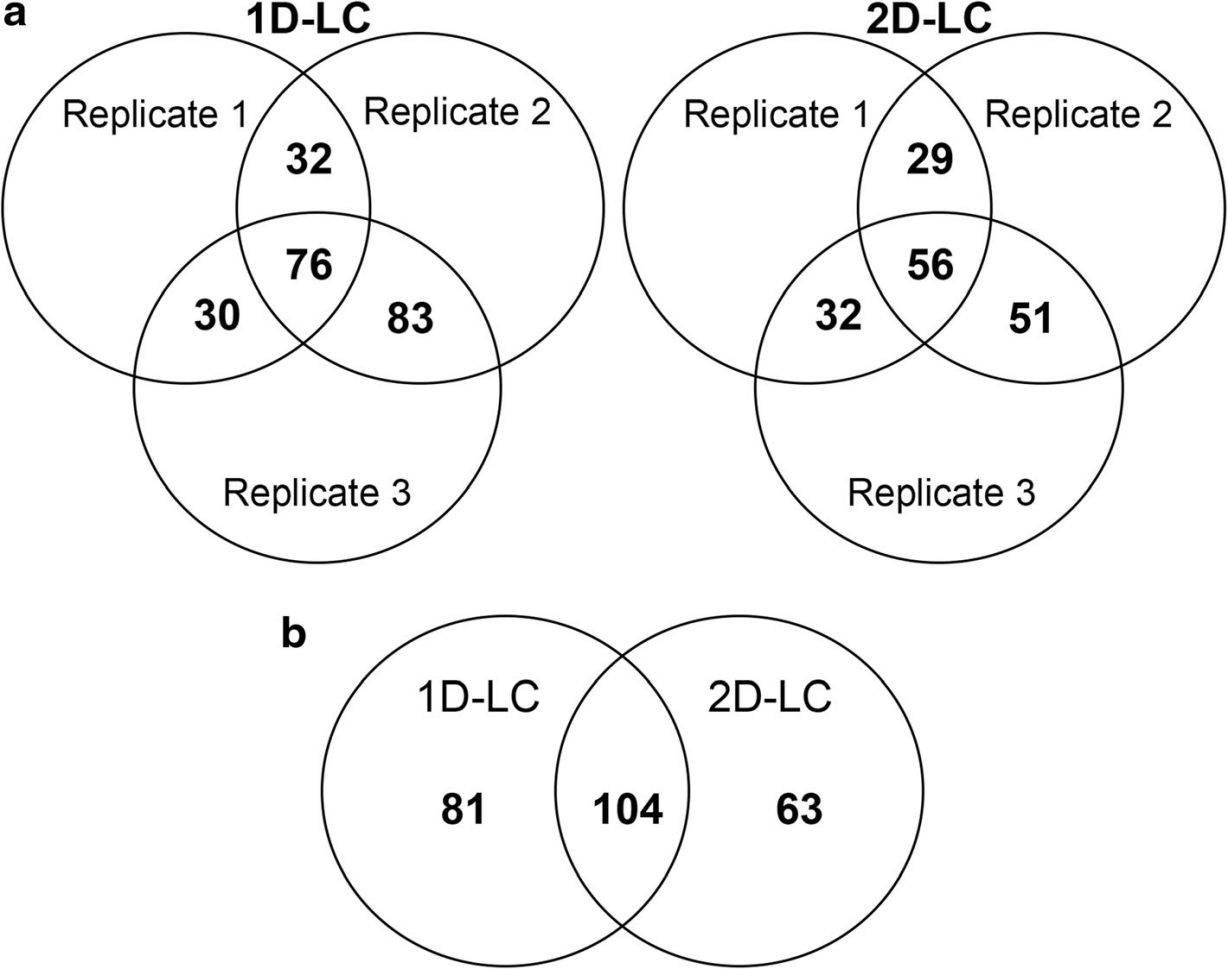
Number of common proteins between 1D-LC and 2D-LC analysis. **a** Number of common proteins with *P* < 0.05 among different biological replicates for both 1D-LC and 2D-LC analysis. **b** Number of common proteins with *P* < 0.05 between 1D-LC and 2D-LC analysis considering 248 proteins included in the study

For comparison between the two pH conditions, fold-changes were calculated and a *p*-value <0.05 was considered significant (Table 1). The distribution of proteins with *P* < 0.05 among the three biological replicates for both 1D-LC and 2D-LC is shown in Fig. 2a. In total, we included 248 proteins found in at least two of the three biological replicates for the biological interpretation. Out of the 248 proteins, 104 were found in both the 1D-LC and the 2D-LC analysis, whereas 81 were uniquely found in the 1D-LC analysis and 63 were uniquely found in the 2D-LC analysis (Fig. 2b).

### Growth in alkaline conditions induce specific changes in the proteome

The identified proteins were analyzed for up- and down-regulation. In general, equal numbers of proteins and similar up- and down-regulation patterns were determined using both 1D-LC and 2D-LC. The identified proteins were grouped according to functionality and were divided into eight different categories: amino acid catabolism and transport, biosynthesis, envelope and periplasmic proteins, proton and electron transport, ribosomal, stress response, sugar catabolism and TCA cycle, and, transcription and translation. Sixty-three proteins were not grouped since most of them belong to putative or uncharacterized proteins.

**Fig. 3:**
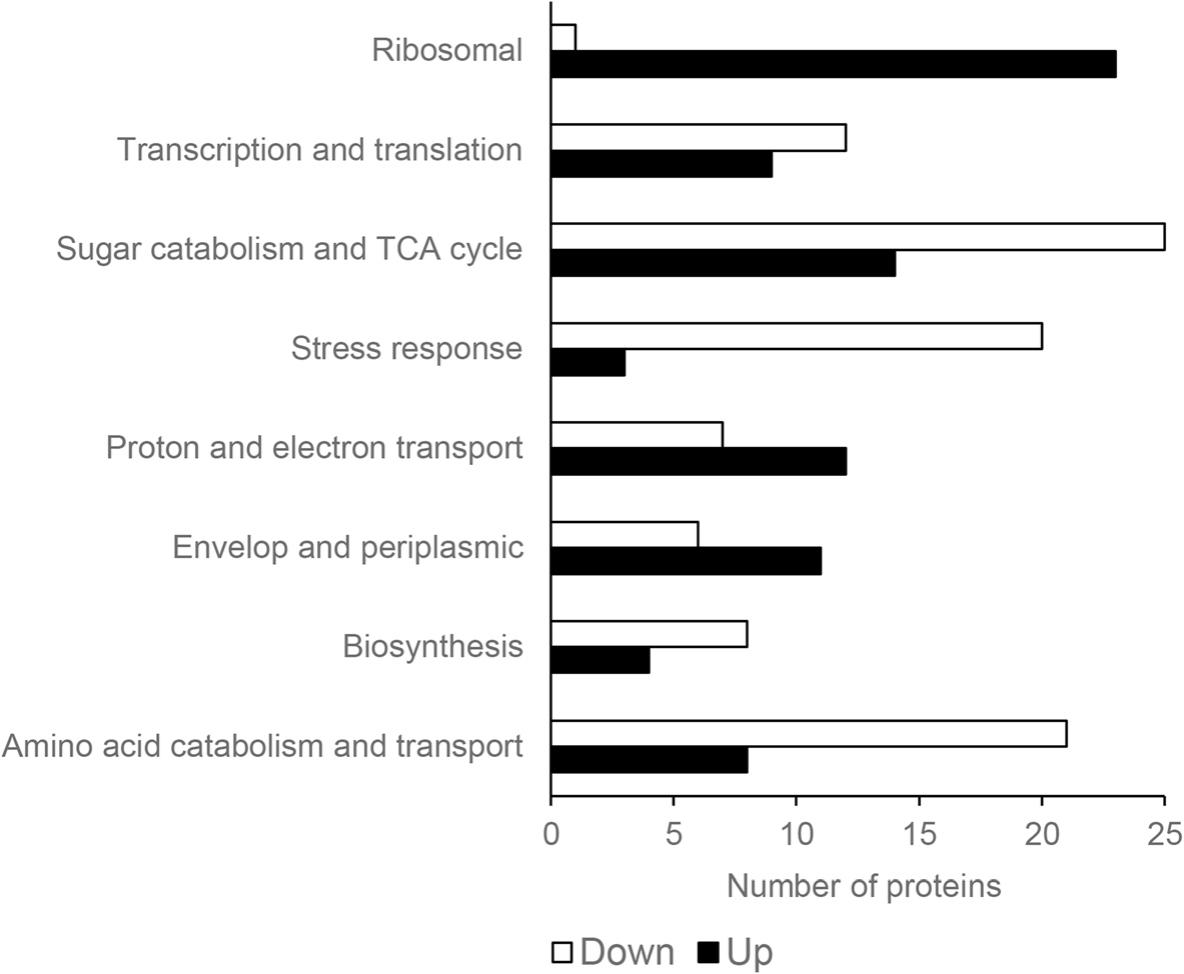
Distribution of up- and down-regulated proteins among the different protein categories

We observed that identified proteins that could be grouped into the categories transcription and translation, ribosomal, proton and electron transport and periplasmic proteins were generally up-regulated under alkaline conditions compared to pH 7. In contrast most of the proteins from sugar catabolism and TCA cycle, stress response, and amino acid catabolism were mainly down-regulated under alkaline conditions (Fig. 3).

Among proteins with the highest fold changes, glutamate decarboxylase A and B (GadAB), pyruvate oxidase (PoxB), L-asparaginase (AnsB) and nitrate reductase (NarH) were the most down-regulated proteins at pH 9 compared to pH 7; whereas proteins belonging to the ATP synthase complex (AtpADFGH) were highly up-regulated. Three uncharacterized proteins were among the most down-regulated proteins at pH 9 (i.e. a hypothetical protein: E8YA36, a putative lipoprotein: E3PFR9, and a putative stress protein: E3PC10). In addition two hypothetical proteins were among the most up-regulated (E8Y559, and E3PLV3 where the latter is predicted to be an exported protein). Although the function of these proteins is unknown, our results suggest that they are involved in alkaline pH responses in *E. coli*.

### Proteins involved in proton and electron transport are up-regulated at alkaline pH

**Fig. 4:**
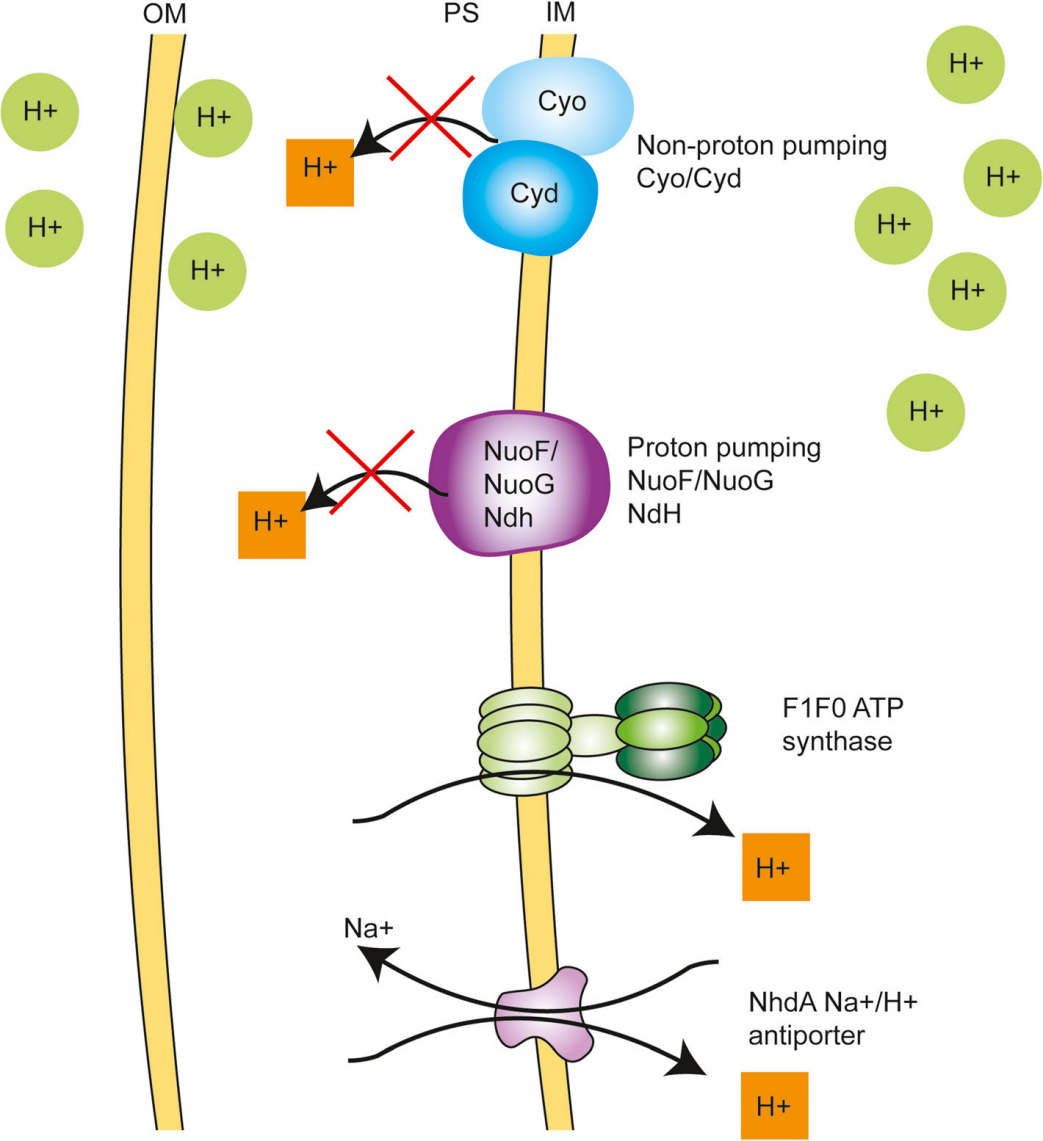
Proton and electron transport system under alkaline conditions. In our system F1Fo ATP synthase, which import H^+^ (orange) to the cytosol during oxidative respiration is up-regulated whereas proton pumping proteins NuoF, NuoG and Ndh are downregulated

We observed strong up-regulation of the ATP synthase operon encoding F1Fo ATP synthase, which import H^+^ to the cytosol during oxidative respiration [20] in contrast to down-regulation of proteins involved in the export of H^+^ from the cytosol such as NADH ubiquinine oxireductase (NuoABCDEFGHI), nitrate reductase A (NarH) and NAD(P)H dehydrogenase (quinone)(WrbA) (Table 2) (Fig. 4). The protein subunit of nitrate reductase (NarH) and dimethyl sulfoxide reductase (DmsA/C) involved in the anaerobic respiration pathway were also down-regulated (Table 2). Furthermore, we observed an increase of Phage shock protein A (PspA) (Table 6), which helps maintain the proton motive force under stress conditions as well as cellular growth during alkaline and nutrient depleted environmental conditions [21]. The proteome at pH 9 thus reflects that several membrane and periplasmic proteins are involved in retaining protons in the cytosol in order to keep a near-neutral pH in the cytosol at alkaline external conditions (Table 2) (Fig. 4).

**Table 2:** Proteins involved in proton and electron transport

Protein	Description	1D-LC	2D-LC	Regulation	Run	Matching
AtpA	ATP synthase subunit alpha	1.93	2.14	UP	All	Both
AtpC	ATP synthase epsilon chain		1.75	UP	2-3	Both
AtpD	ATP synthase subunit beta	1.92	2.14	UP	All	Both
AtpF	ATP synthase subunit b	2.45	3.65	UP	2-3	Both
AtpG	ATP synthase gamma chain	2.12	2.82	UP	All/1-2	Both
AtpH	ATP synthase subunit delta	2.11		UP	2-3	Both
Cmk	Cytidylate kinase		1.60	UP	1-3	Both
DmsA	Anaerobic dimethyl sulfoxide reductase subunit A		0.29	DOWN	2-3	Both
DmsC	Anaerobic dimethyl sulfoxide reductase chain A		0.29	DOWN	2-3	H10407
NarH	Nitrate reductase, beta subunit		0.21	DOWN	All	Both
NirB	Nitrite reductase (NAD(P)H), large subunit	1.69		UP	2-3	Both
NuoF	NADH-quinone oxidoreductase subunit F	0.38		DOWN	2-3	Both
NuoG	NADH-quinone oxidoreductase subunit G	0.63	0.52	DOWN	2-3	Both
SapE	ABC transporter ATP-binding protein	1.67		UP	2-3	H10407
SapF	ABC transporter, ATP-binding protein		1.73	UP	2-3	H10407
TrxA	Thioredoxin	1.68		UP	1-2	H10407
TrxC	Thioredoxin-like protein	1.56		UP	2-3	H10407
WrbA	NAD(P)H dehydrogenase	0.23	0.37	DOWN	All/2-3	Both
YhjA	Cytochrome C peroxidase		0.55	DOWN	1-3	Both

Fold changes are listed under 1D-LC and 2D-LC columns

### TCA cycle proteins are generally down-regulated at alkaline pH while maltose sugar catabolism is favored

**Fig. 5:**
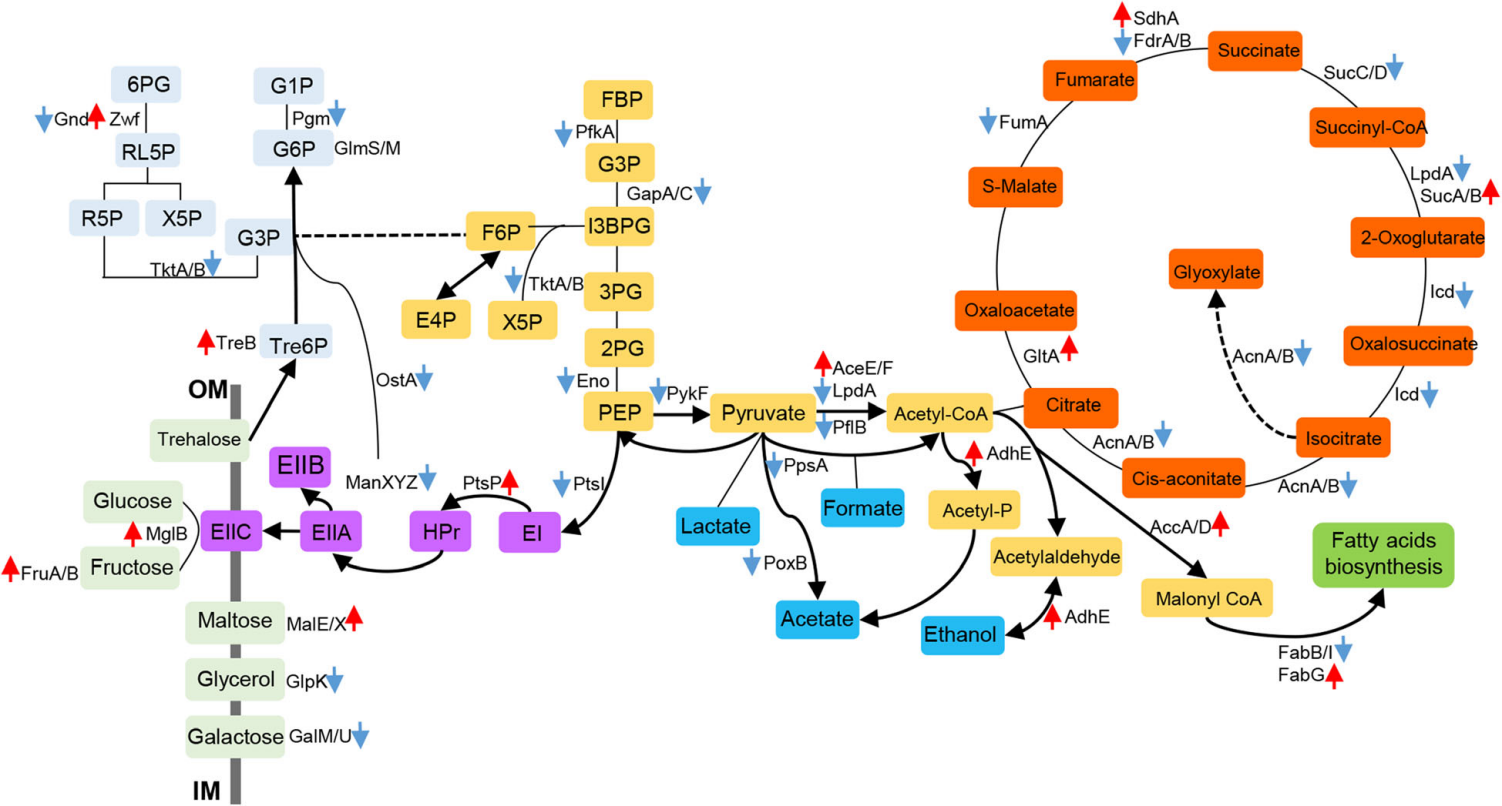
Schematic representation of the sugar catabolism system and TCA cycle under alkaline conditions

The first step in the metabolism of carbohydrates is the transport of these molecules into the cytosol. Substrates need to be transported into cells prior to their catabolic breakdown or employment for anabolic purposes. In bacteria, various carbohydrates are taken up by several mechanisms [22]. The most important transport system for carbohydrates, in particular glucose, is the phosphotransferase system (PTS). All identified enzymes of the PTS system (*e.g.* PtsI, PykF, Pps, PpsA and the Man system) were down-regulated (Table 3). Contrary, the proteins for maltose transport (MalEKMK) and trehalose-specific transporter (TreB) were up-regulated. Expression of genes involved in maltose or maltodextrine transport peak at exponential phase and induction of the maltose operon at alkaline pH has been reported in several studies [23]. It is also known that *E. coli* growing on LB utilize maltose as a preferred carbon source followed by e.g. mannose, melibiose, galactose, fucose and rhamnose [24]. In line with this the galactose/glucose import protein D-galactose-binding periplasmic protein (MglB), a periplasmic binding component of the galactose ABC transporter which is activated in response to low levels of glucose, was up-regulated, implying transport of galactose into the cell. The glucose molecule transported by MglB system is phosphorylated and converted to G6P fructose, which is then transferred and phosphorylated by the fructose PTS (EIIBCFru) system, which was up-regulated at alkaline pH. However, other sugar transport proteins like Glycerol kinase (GlpK), involved in glycerol uptake, and UTP-glucose-1-phosphate uridylyltransferase (GalU) for galactose transport were down-regulated (Fig. 5).

**Table 3:** Proteins involved in sugar catabolism and TCA cycle

Protein	Description	1D-LC	2D-LC	Regulation	Run	Matching
AceE	Pyruvate dehydrogenase E1 component	0.62		DOWN	2-3	Both
AceF	Pyruvate dehydrogenase complex dihydrolipoamide acetyltransferase	1.64	1.65	UP	2-3/1-3	Both
AcnA	Aconitate hydratase	0.44		DOWN	All	Both
Eno	Enolase	0.60		DOWN	1-2	Both
FrdA	Fumarate reductase flavoprotein subunit	0.39	0.35	DOWN	All	Both
FrdB	Fumarate reductase iron-sulfur subunit	0.44	0.36	DOWN	All/1-2	Both
FruB	Bifunctional PTS system fructose-specific transporter subunit IIA/HPr protein	1.89		UP	2-3	Both
FumA	Fumarate hydratase FumB	0.26		DOWN	2-3	Both
GalU	UTP--glucose-1-phosphate uridylyltransferase subunit GalU	0.65	0.55	DOWN	All	Both
GapA	Glyceraldehyde-3-phosphate dehydrogenase A		1.36	UP	1-2	H10407
GapC	Glyceraldehyde-3-phosphate dehydrogenase	0.30	0.27	DOWN	2-3/All	Both
GlpK	Glycerol kinase	0.31	0.31	DOWN	All	Both
GltA	Citrate synthase	1.62	1.74	UP	2-3/All	Both
Gnd	6-phosphogluconate dehydrogenase, decarboxylating	0.43	0.52	DOWN	2-3	Both
GpmA	2,3-bisphosphoglycerate-dependent phosphoglycerate mutase	1.70	1.78	UP	2-3	Both
Icd	Isocitrate dehydrogenase [NADP]		0.87	DOWN	All	Top
MaeA	NAD-dependent malic enzyme	0.62		DOWN	All	H10407
MaeB	Bifunctional malic enzyme oxidoreductase/phosphotransacetylase	2.07		UP	2-3	Both
MalE	Extracellular solute-binding protein family 1	2.03	2.50	UP	All	Both
MalK	Maltose/maltodextrin transporter ATP-binding protein	2.03		UP	1-3	Both
MalM	Maltose operon periplasmic	1.85	1.88	UP	All/2-3	Top
MalX	Maltose transport system, substrate-binding protein	2.03	2.50	UP	All	H10407
ManXYZ	PTS system mannose-specific transporter subunits IIAB	0.69		DOWN	1-3	Top
MglB	Methyl-galactoside ABC transporter galactose-binding periplasmic protein		3.70	UP	1-2	Both
PfkA	6-phosphofructokinase	0.48	0.41	DOWN	All/2-3	Both
PflB	Pyruvate formate lyase I	0.42	0.36	DOWN	1-2	Both
Pgm	Phosphoglucomutase		0.43	DOWN	1-3	Both
PoxB	Pyruvate dehydrogenase	0.28	0.08	DOWN	All/2-3	Both
Pps	Phosphoenolpyruvate synthase	0.62	0.60	DOWN	All/1-2	Top
PpsA	Phosphoenolpyruvate synthase	0.62	0.60	DOWN	All/1-2	H10407
PtsI	Phosphoenolpyruvate-protein phosphotransferase		0.95	DOWN	All	Both
PykF	Pyruvate kinase	0.54	0.41	DOWN	All	Both
SucA	Succinyl-CoA ligase [ADP-forming] subunit alpha	0.52	0.45	DOWN	All	H10407
SucC	Succinyl-CoA ligase [ADP-forming] subunit beta	0.39		DOWN	2-3	Both
SucD	Succinyl-CoA ligase [ADP-forming] subunit alpha	0.56	0.45	DOWN	1-2/All	Top
TktA	Transketolase	0.52	0.47	DOWN	All	Both
TktB	Transketolase	0.52	0.49	DOWN	2-3/1-2	Both
TreB	PTS system trehalose(Maltose)-specific transporter subunits IIBC	2.01	2.07	UP	2-3/All	Both
Zwf	Glucose-6-phosphate 1-dehydrogenase	2.23	2.54	UP	2-3	Both

Fold changes are listed under 1D-LC and 2D-LC columns

The enzymes of the glycolytic pathway, the pentose phosphate pathway and TCA cycle were generally down-regulated under alkaline conditions (Fig. 5). Acetate formation through pyruvate dehydrogenase (PoxB), and lactate formation through D-lactate dehydrogenase (LdhA) and phosphoenoloyruvate synthase (PpsA), which catalyzes conversions from pyruvate to PEP, does not seem to play a significant role under alkaline conditions since these proteins were down-regulated. In contrast, glucose-6-phosphate 1-dehydrogenase G6PDH (Zwf), which is a key enzyme in central metabolism was up-regulated. G6PDH is involved in the distribution of carbon between glycolysis and the pentose phosphate pathway (PPP), which provides a large portion of the NADPH needed for anabolism. But G6PDH is also activated in response to oxidative stress by the soxRS regulatory system [25, 26].

Most identified enzymes belonging to the pentose phosphate pathway (e.g. Gnd, TktA) have previously been found in lower amounts in cells growing under alkaline conditions [27]. We observed down-regulation of both transketolase A and B (TktA, TktB) involved in the nonoxidative branch of the pentose phosphate pathway in contrast to other studies where TktA and TktB have been suggested to be regulated in opposite ways, for instance the TktB gene is induced while TktA is repressed by RpoS [28].

### Periplasmic and outer membrane protein transport over membranes is up-regulated at alkaline conditions

ETEC toxin secretion has been shown to be favored by alkaline pH [7]. We hypothesized that alterations in the composition of proteins at the membrane and periplasmic level allows for higher secretion of LT toxin. The Sec machinery mediates translocation of LT toxin A and B subunits across the inner membrane in a process that is dependent on ATP and the proton motive force. In line with this up-regulation of the Sec translocation complex (SecD/F) was observed (Table 4), In addition upregulation of YidC was also observed. YidC is an integral membrane chaperone that interacts transiently with membrane proteins during their biogenesis and stimulates their correct assembly [29]. YidC interacts with SecD and SecF to form a heterotetrameric SecDFYajCYidC accessory complex [30].

**Table 4:** Envelop and periplasmic proteins

Protein	Description	1D-LC	2D-LC	Regulation	Run	Matching
AcrA	Acriflavin resistance protein A	2.54	2.78	UP	2-3/1-3	Both
BamA	Outer membrane protein assembly factor BamA		1.50	UP	1-2	H10407
BamD	Outer membrane protein assembly factor BamD		1.55	UP	All	Both
CopA	Copper exporting ATPase		0.44	DOWN	2-3	Both
DsbA	Thiol:disulfide interchange protein	2.33	2.00	UP	All/2-3	Both
HchA	Molecular chaperone Hsp31 and glyoxalase 3		0.41	DOWN	All	Both
OmpA	Outer membrane protein A		1.60	UP	2-3	Top
SecB	Protein-export protein SecB	0.29	0.29	DOWN	All	Both
SecD	Protein translocase subunit SecD	1.67		UP	All	Both
SecF	Protein-export membrane protein SecF		1.77	UP	2-3	Both
SlyB	Outer membrane lipoprotein SlyB	1.54		UP	1-2	Top
TraT	Putative complement resistance protein TraT	0.52		DOWN	1-3	H10407
YbiT	ABC transporter ATP-binding protein	1.67		UP	2-3	Top
YbjP	Putative lipoprotein	0.52	0.46	DOWN	1-2/2-3	Top
YidC	Membrane protein insertase YidC	2.04	1.86	UP	All	Both
YjjK	ABC transporter related protein		1.34	UP	All	Top
YtfQ	Periplasmic binding protein/LacI transcriptional regulator	0.38		DOWN	2-3	Top

Fold changes are listed under 1D-LC and 2D-LC columns

In the periplasmic space the LT toxin is assembled in a pH- and DsbA-dependent manner and secreted through the general type II secretion pathway. Since we observed up-regulation of DsbA it is possible that increased assembly of LT holotoxin in the periplasm can explain elevated levels of secretion of LT toxin at high pH. The Gsp components of the type II secretion pathway were however not significantly changed consistent with other findings [31].

The β-Barrel Assembly Machinery complex (BamAD) that is essential for insertion of outer membrane proteins (OMPs) in the outer membrane of gram-negative bacteria was up-regulated, in line with this the chaperone SurA that escorts outer membrane proteins to the Bam complex was induced (Table 6) as well as the outer membrane protein OmpA (Table 4). Hence, alkaline conditions might favor expression of outer membrane proteins and/or secretion in general.

### The osmotic stress responses are generally down-regulated at alkaline pH

In response to pH stress *E. coli* respond with different adaptive mechanisms including induction of pH dependent chaperones and osmoprotectants. We found that proteins involved in acidic stress response, *i.e.* GadAB and the acid stress induced chaperone HdeB were down-regulated as expected. In addition, trehalose-6-phosphate synthase OtsA that synthesizes the osmoprotectant trehalose under osmotic stress was down-regulated (Table 5). Additionally, two osmotically regulated permeases, ProP and ProU involved in the uptake of osmoprotectant molecules such as glycine betaine and proline were down-regulated. The osmotically induced proteins OsmB/E/Y were also down-regulated [32] (Table 5). Taken together this indicates that alkaline stress is reducing osmotic stress responses in *E. coli.*

**Table 5:** Proteins involved in biosynthesis and stress response

Protein	Description	1DLC	2DLC	Regulation	Run	Matching
AccB	Acetyl-CoA carboxylase biotin carboxyl carrier	1.83		UP	1-3	Both
AccD	Acetyl-coenzyme A carboxylase carboxyl transferase	1.53	1.93	UP	2-3	Both
ClpA	ATP-dependent Clp protease ATP-binding subunit	0.66		DOWN	1-2	H10407
ClpB	ATP-dependent chaperone ClpB	0.50	0.48	DOWN	1-2/All	Top
CspE	Cold shock protein CspE	1.72	1.74	UP	2-3/1-2	Both
DdlA	D-alanine--D-alanine ligase		0.44	DOWN	1-3	Both
DnaK	Chaperone protein DnaK	0.63	0.59	DOWN	All	Both
ErfK	ErfK/YbiS/YcfS/YnhG family protein	0.64		DOWN	2-3	Top
FabB	3-oxoacyl-(Acyl carrier protein) synthase I	0.59	0.49	DOWN	All	Both
FabF	3-oxoacyl-[acyl-carrier-protein] synthase 2	0.61		DOWN	2-3	Both
GadA	Glutamate decarboxylase	0.18	0.06	DOWN	1-2/2-3	Both
GadB	Glutamate decarboxylase beta subunit	0.08	0.04	DOWN	All/2-3	H10407
GlmM	Phosphoglucosamine mutase	0.64		DOWN	1-3	Top
GlmS	Glutamine--fructose-6-phosphate aminotransferase		0.61	DOWN	All	Both
GlnS	Glutamine--tRNA ligase	0.52		DOWN	2-3	Both
Gmk	Guanylate kinase	0.42		DOWN	2-3	Both
GrcA	Autonomous glycyl radical cofactor	0.41	0.32	DOWN	All	H10407
GroEL	60 kDa chaperonin	2.62	2.79	UP	2-3	Both
GshB	Glutathione synthetase		0.46	DOWN	2-3	Both
HdeB	Acid stress chaperone HdeB	0.22		DOWN	2-3	Both
HdhA	7-alpha-hydroxysteroid dehydrogenase	0.27		DOWN	2-3	Both
HtpB	Chaperone protein HtpG		0.74	DOWN	1-3	Top
KatG	Catalase-peroxidase		1.90	UP	2-3	Both
LpxA	Acyl-UDP-N-acetylglucosamine O-acyltransferase	1.51		UP	2-3	Top
MsyB	SecY/secA suppressor protein	0.58		DOWN	2-3	Both
NapA	Periplasmic nitrate reductase	0.46		DOWN	1-3	Both
OsmB	Osmotically inducible lipoprotein E	0.46	0.36	DOWN	1-3/All	H10407
OsmE	DNA-binding transcriptional activator OsmE	0.46	0.36	DOWN	1-3/All	Top
OsmY	Osmotically-inducible protein Y	0.28	0.26	DOWN	All	Both
OtsA	Alpha,alpha-trehalose-phosphate synthase	0.67		DOWN	1-2	Both
Prs	Ribose-phosphate pyrophosphokinase		1.60	UP	2-3	Top
PurA	Adenylosuccinate synthetase	0.64	0.62	DOWN	All/2-3	Both
Skp	Chaperone protein skp	0.59		DOWN	2-3	Both
SspA	Glutathione S-transferase domain protein	0.66	0.55	DOWN	1-3	Both
YbaY	Glycoprotein/polysaccharide metabolism	0.21		DOWN	2-3	Top

Fold changes are listed under 1D-LC and 2D-LC columns

The heat shock response is one of the main global regulatory networks in all organisms and involves an increased cellular level of chaperones and proteases to enable correct protein folding and balanced growth under different stress conditions [33]. The heat shock response in *E. coli* is mediated by σ32 [33]. Among the heat shock proteins that passed our criteria for changed expression we found that DnaK and ClpAB were repressed in response to alkaline stress. These results were in contrast with earlier findings that have indicated that DnaK is induced by alkaline conditions [34] but supported by findings of Maurer et al. [35]. We found that the heat shock protein GroEL was up-regulated consistent with other reports [34]. We also found that the cold shock protein E CspE was up-regulated. Among proteases, DegP was up-regulated and PepD was down-regulated. DegP degrades abnormal proteins in the periplasm, including mutant proteins, oxidatively damaged proteins and aggregated proteins [36] (Table 5).

### Translation and transcription is induced at alkaline pH

Proteins involved in translation were mainly up-regulated such as ribosomal proteins, ribosomal associated proteins, elongation factors, peptide chain release factor PrfC, RNA degradation protein Pnp and the initiation factor InfB. InfB was been previously reported to be produced in response to stress conditions since accumulation of InfB seems to be correlated with a stop of protein synthesis in *E. coli* [37]. These results suggest an increased production of proteins in response to alkaline stress or alternatively an increased re-localization of ribosomes to the inner membrane.

Under alkaline stress, RNA polymerase proteins RpoA/B/C were up-regulated suggesting that transcription was favored. Among transcriptional factors, the global regulators ArcA, IhfA/B and Crp were down-regulated (Table 6). Proteins involved in ATP generation in the absence of oxygen or other electron acceptors, which are positively regulated by the transcriptional regulator ArcA, *i.e.* Ppc involved in succinate formation, and PflB in the pyruvate dehydrogenase complex (PDHc), were down-regulated, in contrast to up-regulation of the AceE/F complex which encode *α* and *β* subunits of PDHc and is negatively regulated by ArcA. These results were consistent with the observed down-regulation of ArcA itself and indicate that alkaline pH repress the ArcA regulon. RpoS responsible of the expression of many genes under stress conditions was not detected in our analysis.

**Table 6:** Proteins involved in transcription and translation mechanisms

Protein	Description	1D-LC	2D-LC	Regulation	Run	Matching
ArcA	Aerobic respiration control protein	0.63		DOWN	All	Both
Crp	Cyclic AMP receptor protein		0.65	DOWN	1-3	Both
DegP	Protease Do	1.82	2.16	UP	All	Top
Dps	DNA protection during starvation protein	0.39	0.28	DOWN	2-3	Both
GreA	Transcription elongation factor GreA	1.89		UP	2-3	Both
HflC	Modulator of FtsH protease HflC	2.01	1.89	UP	1-3/All	H10407
HflK	FtsH protease regulator HflK	1.60	2.27	UP	1-3/All	Both
HflX	GTPase HflX	1.60	2.27	UP	1-3/All	H10407
IhfA	Integration host factor subunit alpha	0.62	0.63	DOWN	All/2-3	Both
LepA	Elongation factor 4		1.65	UP	All	Both
LepB	Signal peptidase I		1.68	UP	1-2	Both
NrdA	Ribonucleoside-diphosphate reductase	0.53	0.57	DOWN	2-3/All	Both
NrdD	Anaerobic ribonucleoside triphosphate reductase	0.56		DOWN	2-3	Top
NusA	NusA antitermination factor	0.63		DOWN	2-3	Top
PepD	Aminoacyl-histidine dipeptidase	0.64		DOWN	1-2	Top
Pnp	Polyribonucleotide nucleotidyltransferase	1.58	1.78	UP	All	Both
PolA	DNA polymerase I		0.65	DOWN	1-2	Top
PrfC	Peptide chain release factor 3		1.54	UP	1-2	Both
PspA	Phage shock protein A	1.69	2.27	UP	All/2-3	Both
RaiA	Sigma 54 modulation protein/ribosomal	1.55		UP	2-3	Top
Rho	Transcription elongation protein	0.63		DOWN	2-3	H10407
RlpK	50S ribosomal protein L11	1.54	1.78	UP	1-3	Both
Rph	Ribonuclease PH	1.67	2.04	UP	All/2-3	Both
RplC	50S ribosomal protein L3	1.54	1.64	UP	All/1-3	Both
RplD	50S ribosomal protein L4	1.31		UP	1-3	Top
RplF	50S ribosomal protein L6	1.28		UP	1-3	Top
RplI	50S ribosomal protein L9	1.70	1.87	UP	All/2-3	Both
RplK	50S ribosomal protein L11	1.53	1.78	UP	1-3	Top
RplO	50S ribosomal protein L15		1.87	UP	1-3	Both
RplT	50S ribosomal protein L20	1.73	1.86	UP	All	Both
RpmE	50S ribosomal protein L31	1.98		UP	All	Both
RpmG	50S ribosomal protein L33		1.95	UP	1-3	Both
RpoA	DNA-directed RNA polymerase subunit alpha		1.66	UP	2-3	Both
RpoB	DNA-directed RNA polymerase subunit beta	1.84	1.66	UP	2-3/All	Both
RpoC	DNA-directed RNA polymerase subunit beta’	1.92	1.71	UP	2-3/All	Both
RpsA	30S ribosomal protein S1		1.40	UP	1-3	H10407
RpsH	30S ribosomal protein S8		1.69	UP	2-3	Both
RpsJ	30S ribosomal protein S10	1.52		UP	1-3	Top
RpsK	30S ribosomal protein S11		2.53	UP	All	Both
RpsO	30S ribosomal protein S15	1.52	1.53	UP	2-3	H10407
Sra	Stationary phase induced ribosome associated protein	0.55		DOWN	2-3	H10407
Ssb	Single-stranded DNA-binding protein	1.80	1.95	UP	1-2/2-3	Both
SurA	Chaperone SurA		1.82	UP	1-3	Both
UspF	Universal stress protein F	0.43	0.42	DOWN	2-3/All	Top
YgfZ	tRNA-modifying protein YgfZ		0.52	DOWN	1-3	Both

Fold changes are listed under 1D-LC and 2D-LC columns

### Amino acid metabolism under alkaline conditions

High pH have been reported to induce enzymes involved in generation of ammonia from amino acids including TnaA, CysK and AstD which consume tryptophan, serine, cysteine and arginine. Our results indicated a down-regulation in amino acid metabolism. TnaA, tryptophan deaminase, was down-regulated under alkaline conditions (Table 7). This result does not match with previous studies where TnaA was favored by alkaline pH [23]. TnaA has been reported to act as an important signaling molecule during alkaline conditions and to be regulated by RpoS under different environmental or growth conditions [38]. RpoS regulated proteins have a more important role in extended periods of stationary phase growth rather than at the onset of stationary phase [39]. It is possible that TnaA was down-regulated in our system, since the analysis of proteins was done during exponential phase. Furthermore, PutA required for the degradation of proline was up-regulated. In the presence of proline, PutA is associated with the cytoplasmic membrane and acts a bifunctional enzyme catalyzing both reactions of the proline degradation pathway: the oxidation of proline by proline dehydrogenase and subsequent oxidation to glutamate by pyrroline-5-carboxylate (P5C) dehydrogenase.

**Table 7:** Proteins involved in amino acid catabolism and transport

Protein	Description	1D-LC	2D-LC	Regulation	Run	Matching
AlaS	Alanine--tRNA ligase	0.65		DOWN	2-3	H10407
AnsB	L-asparaginase II	0.20		DOWN	2-3	Top
AroK	Shikimate kinase 1		1.70	UP	2-3	Both
AsnA	Aspartate--ammonia ligase	0.54		DOWN	2-3	Both
AsnS	Asparagine--tRNA ligase	0.63	0.63	DOWN	2-3/All	Both
AspA	Aspartate ammonia-lyase	0.52	0.50	DOWN	All/1-3	Both
AspS	Aspartate--tRNA ligase	1.56	2.28	UP	All	Both
CarA	Carbamoyl-phosphate synthase small chain		2.53	UP	1-2	Both
CarB	Carbamoyl-phosphate synthase large chain	3.00	4.01	UP	2-3/All	Both
CysI	Sulfite reductase hemoprotein beta-component		0.47	DOWN	1-3	Both
GabT	4-aminobutyrate aminotransferase	0.39		DOWN	2-3	Both
IleS	Isoleucine--tRNA ligase	0.52	0.62	DOWN	2-3/All	Both
IlvC	Ketol-acid reductoisomerase	1.79		UP	1-2	Top
Klb	2-amino-3-ketobutyrate coenzyme A ligase	0.51		DOWN	1-2	Both
LeuS	Leucine--tRNA ligase		0.46	DOWN	2-3	Both
LysS	Lysine--tRNA ligase	0.44	0.40	DOWN	All	Both
LysU	Lysine--tRNA ligase	0.47	0.38	DOWN	All	Both
MetG	Methionine--tRNA ligase	0.71		DOWN	1-3	Top
MetK	S-adenosylmethionine synthase	0.57	0.36	DOWN	All	Both
PheS	Phenylalanine--tRNA ligase alpha subunit		1.50	UP	1-2	Both
ProP	Metabolite/H+ symporter, major facilitator superfamily	0.40	0.38	DOWN	1-2	Both
ProQ	ProP effector		1.87	UP		Both
ProV	Glycine betaine transporter ATP-binding subunit	0.55	0.63	DOWN	2-3	Both
PutA	Delta-1-pyrroline-5-carboxylate dehydrogenase		1.61	UP	1-3	Both
SerS	Serine--tRNA ligase	0.50	0.45	DOWN	All/2-3	Both
Tdh	L-threonine 3-dehydrogenase		0.57	DOWN	2-3	Both
TnaA	Tryptophanase	0.34		DOWN	1-2	Both
TyrS	Tyrosine--tRNA ligase	0.54	0.60	DOWN	1-2	Both
ValS	Valine--tRNA ligase		0.66	DOWN	1-3	Top

Fold changes are listed under 1D-LC and 2D-LC columns

## Discussion

Our study highlights the effect of alkaline pH on the expression of proteins compared to neutral pH in ETEC. Since we hypothesize that infecting ETEC might encounter alkaline pH close to the epithelium the alkaline surface proteome might provide a better view of ETEC behavior at the site of infection and aid in identification of *e.g.* novel vaccine targets. However, even if our study is focused on ETEC, we have also identified general mechanisms based on the MS results that could be extrapolated to all *E. coli* subspecies.

The LC-MS/MS method used generated results for 3–400 proteins at each condition while *E. coli* is expected to express around 3000 genes of which most are translated into proteins. In this study we tried to enrich for the surface and membrane proteome, which is estimated to comprise 40% of the bacterial total proteome [40]. Each generated sample was split into two parts and subsequently two different analysis approaches were employed, here named 1D-LC and 2D-LC. This was done in order to maximize the number of identified peptides generated by the enzymatic digestion. It has been estimated that for highly complex samples, containing 10,000–50,000 proteins in different concentration ranges, theoretically it would be necessary to be able to separate around 10% of the peptides prior to MS analysis [41]. Normally, 1D-LC approaches are not able to resolve this number of peptides, and thus multidimensional separation strategies such as 2D-LC analysis was employed [42] to maximize the number of peptides/protein identifications.

As we used the surface shaving approach, the samples were less complex relative to a whole cell lysate, where orthogonal fractionation would be necessary. The lower complexity and the use of long separation gradients with two exclusion lists enabled the 1D separation to perform well. For a sample of low complexity, using the 2D approach will not always be beneficiary since there is a risk that some peptides, particularly of low abundance, might be lost in the first offline fractionation step. This might explain the reason for the data being complementary to each other when comparing the 1D-LC vs 2D-LC approach.

The two methods employed generated slightly different results. In previous shotgun proteomics studies, 1D-LC with 2D-LC approaches have been compared [41, 43]. Concerning the performance on 2D-LC approaches, the drawback of having to use offline fractionation (SCX or RP) prior to a second online RP-LC injection of the fractions, is balanced against a higher number of peptide/protein identifications. When comparing the methodologies on the same sample, most of the identified proteins in a 1D-LC set-up are usually found also in the 2D-LC set-up [41]. In our study, however, the employed 1D-LC (RP-LC) set-up of three consecutive injections (with exclusion lists) seemed to complement the 2D-LC set-up (eight SCX fractions followed by RP-LC). The number of proteins found in both set-ups was 104 whereas 81 proteins were uniquely found in the 1D-LC set-up versus 63 proteins uniquely found in the 2D-LC set-up. This might be due to the methodology of generating the peptides, using the LPI methodology to perform surface shaving of intact bacteria. Also, it seems that hydrophobic peptides might be underrepresented when using SCX as the first dimension of analysis [41]. Further studies would be needed to explain why our approach showed that the 1D-LC and 2D-LC set-ups provided complimentary data instead of having a more overlapping character, this was however not the scope of this analysis.

Growth at pH 9 poses a specific type of stress where the bacteria needs to pump H^+^ into the cytosol to maintain a near neutral intracellular pH homeostasis, needed for normal function. Under pH stress conditions, *E. coli* needs to maintain the cytoplasmic pH between 7.2 and 7.8 in order to preserve enzymatic activity and nucleic acid and protein stability [44]. Under alkaline conditions this is executed through active influx of protons and restrained outflux of protons from the cytosol. At aerobic conditions, *E. coli* produce NADH and FADH_2_ through the TCA cycle, these reducing equivalents are oxidized in the respiratory chain, and the electrons generated from the reducing equivalents are subsequently transferred to cytochromes where O_2_ is converted to H_2_O [27]. This process is coupled to the formation of a proton motive force (PMF) over the cytoplasmic membrane, which is utilized for ATP generation from ADP and Pi through the ATP synthase complex. NADH is oxidized in the respiratory chain via a coupled NADH dehydrogenase NDH-1 encoded by *Nuo* and *Ndh,* which export H^+^ to the periplasma while one electron is transported through the respiration system [45].

It is known that at high pH, NhaA, a sodium ion/proton antiporter uses the proton electrochemical gradient to expel sodium ions from the cytoplasm and functions primarily in the adaptation to high salinity at alkaline pH helps to maintain internal pH and to protect cells from excess sodium [46]. However, the mechanisms of regulation of internal pH when bacteria encounters stress conditions and how these conditions are related with virulence it is not well understood. Na^+^/H^+^ exchange would supply intracellular bicarbonate by export of H^+^ formed on hydration of CO_2_ to H^+^ and HCO_3−_ [47]. In line with previous studies on *E. coli*, we confirmed that ETEC prefer ATP synthase for import of protons when alkaline stress occurs on cytoplasmic pH and prefers to minimize proton export associated with NADH-I system including Nuo, Ndh and WrbA proteins as shown in our suggested model system in Fig. 4 [35].

Our findings are largely consistent with previous studies on alkaline conditions in *E coli* [35, 44, 48].

We found that alkaline conditions down-regulate osmotic stress responses and these findings confirm the previously suggested link between acid stress and oxidative stress [35]. Heat-shock inducible genes such as DnaK and ClpAB were down-regulated at alkaline conditions consistent with results by Maurer et al., [35], but we also found induction of GroEL expression. We were however not able to identify alkaline tolerance proteins such us NhaA, NhaB, ChaA, MdtM, and MdfA involved in the exchange of protons for other cations [44, 49], neither the YqjA protein which has recently reported to have proton-dependent transport activity [50].

The results indicate that proteins involved in osmotic and heat-shock regulation were generally down-regulated at alkaline conditions. Secretion of LT toxin has previously been reported to be favored in the presence of salts (NaCl) and high osmolarity (sucrose) [51]. It is possible that alkaline conditions override the need for high osmolarity but since we used buffered LBK media without NaCl in this study, further studies are needed to elucidate the difference between Na^+^ and K^+^ and its impact on ETEC virulence and secretion. In addition, since an acute infection would lead to a massive efflux of Na^+^ and Cl^−^ ions locally from the infected cell analyses on the effect of the infectious microenvironment on ETEC virulence would be very interesting to pursue [11].

The surface proteome analysis of ETEC was performed in order to observe changes in the outer membrane proteome that could provide insight into the surface expression at the site of infection. We were also interested to find an explanation to higher secretion level of the LT toxin under alkaline conditions [7]. We found evidence that alkaline conditions induce the secretome of ETEC since the Sec pathway was up-regulated. Export of LT subunits and several other proteins are targeted through the signal recognition particle pathway to the Sec translocon for transport peptides into the periplasm. YidC interacts with SecYEG through the SecY subunit, but also with SecD and SecF to form a heterotetrameric SecDFYajCYidC accessory complex [29, 30]. Under alkaline pH, YidC is a relatively abundant inner membrane protein [29, 52]. In the Sec associated form, YidC recognizes individual protein transmembrane domains in the context of the Sec translocon early during biogenesis and facilitate their folding [29, 53]. It is hence possible that YidC is involved in specific recognition of peptides secreted during alkaline conditions but this needs to be verified. We also found that the outer membrane BAM complex as well as OmpA were up-regulated under alkaline conditions.

## Conclusions

Based on our results we hypothesize that ETEC adhesion to the epithelium gradually induce the outer membrane proteome and secretome including secretion of the LT toxin due to the increase in local pH caused by bicarbonate secreted by the deregulated CFTR channels of the infected epithelial cells. Our findings indicate that ETEC likely respond to an alkaline microenvironment close to the epithelium during infection by up-regulation of Sec dependent translocation over the inner membrane followed by increased assembly of a specific repertoire of proteins that are secreted and/or associated to the outer membrane. Additional studies are however needed to define the secretome during infection of the human gastrointestinal tract.

## Abbreviations

1D-LCMS: One-dimentional Liquid chromatography–mass spectrometry
2D-LCMS: Two-dimentional Liquid chromatography–mass spectrometry
CT: Cholera toxin
ETEC: Enterotoxigenic *Escherichia coli*
LB: Luria-Bertani media
LPI: Lipid-based protein immobilization
LT: Heat-labile toxin
MS/MS: Tandem mass spectrometry
PMF: Proton motive force
PTS system: Phosphotransferase system
ST: Heat-stable toxin
TCA: Citric acid cycle
TMT: Tandem mass tag
